# Impact of 3D-printed models in meetings with parents of children undergoing interventional cardiac catheterisation

**DOI:** 10.3389/fped.2022.947340

**Published:** 2023-01-09

**Authors:** Clément Karsenty, Khaled Hadeed, Camelia Djeddai, Julie Lateyron, Aitor Guitarte, Remi Vincent, Nathalie DeBarros, Nicolas Combes, Jerome Briot, Yves Dulac, Antoine Yrondi, Philippe Acar

**Affiliations:** ^1^ Department of Paediatric Cardiology, University Hospital, Toulouse, France; ^2^ Institut Des Maladies Métaboliques Et Cardiovasculaires (Institute of Metabolic and Cardiovascular Diseases), University of Toulouse, INSERM U1048, I2MC, 1, Avenue Jean Poulhès-BP84225, Toulouse, France; ^3^ Department of Cardiology, Clinique Pasteur (Pasteur Clinic), Toulouse, France; ^4^ Department of Psychiatry, Marchant Hospital, Toulouse, France; ^5^ Department of Psychiatry, University Hospital, Toulouse, France

**Keywords:** 3D-printed model, catheterisation, anxiety, patient information, complex congenital heart disease

## Abstract

**Background:**

Paediatric interventional catheterisation has consistently improved in recent decades, with often highly successful outcomes. However, progress is still required in terms of the information delivered to parents and how parental anxiety is managed.

**Aim:**

To investigate the impact of cardiac printed models on improving parental understanding and alleviating anxiety before interventional catheterisation.

**Methods:**

The parents of children undergoing interventional cardiac catheterisation were prospectively enrolled in the study. A questionnaire highlighting knowledge and understanding of the condition and cardiac catheterisation per se was scored on a scale of 1–30. The State-Trait Anxiety Inventory (STAI), which generates current anxiety scores, was also used before and after the pre-catheterisation meeting. The “printing group” received an explanation of catheterisation using the device and a three-dimensional (3D) model, while the “control group” received an explanation using only the device and a manual drawing.

**Results:**

In total, 76 parents of 50 children were randomly assigned to a “control group” (*n* = 38) or “printing group” (*n* = 38). The groups were comparable at baseline. The level of understanding and knowledge improved after the “control group” and “printing group” meetings (+5.5±0.8 and +10.2±0.8; *p* < 0.0001 and *p* < 0.0001, respectively). A greater improvement was documented in the “printing group” compared to the “control group” (*p* < 0.0001). The STAI score also improved after the explanation was given to both groups (−1.8±0.6 and −5.6±1.0; *p* < 0.0001 and *p* < 0.0001). The greatest improvement was noted in the “printing group” (*p* = 0.0025). Most of the parents (35/38 from the “printing group”) found the models to be extremely useful.

**Conclusion:**

3D-printed models improve parental knowledge and understanding of paediatric cardiac catheterisation, thereby reducing anxiety levels.

## Introduction

Over the last three decades, dramatic changes have occurred in paediatric cardiac catheterisation, which has evolved from a diagnostic to a therapeutic procedure in the management of congenital heart defects (CHD) (1). Considerable progress has been made with excellent outcomes, but challenges nevertheless remain (2). First, particularly in the paediatric context, the notion of informed consent gives interventional cardiologists the opportunity not only to ensure that patients and relatives alike agree with elective catheterisation but also allow them to inform, educate and prepare patients and their families for the imminent procedure (3, 4). Second, anxiety levels are high among parents of children undergoing cardiac procedures, including catheterisation and surgery (5, 6). Parental anxiety was higher before CHD surgeries than in other paediatric congenital surgeries, thus confirming that this is indeed a stressful experience (7). The greater the level of parental anxiety on the day of the intervention, the more traumatic the experience for their child (8). Hence, improving the psychological well-being of parents is now a matter of priority for healthcare CHD professionals with greater focus on how medical information is delivered to parents.

In a recent pilot study, Boyer et al. found that pre-catheterisation consultations, including angiograms and three-dimensional (3D) printed cardiac models, reduce the anxiety of patients and their families before a procedure (9).

3D-printed cardiology applications range from improving diagnostic work-up to guiding treatment strategies, simulating interventional and surgical procedures, and enhancing teaching (10–13). 3D models can also help patients and their families to improve their understanding of underlying CHD anatomy and the need for a procedural intervention. For instance, 3D-printed patient-specific models of CHD have improved both patient engagement and physician–parent–patient communication in clinical practice (14).

Thus, we investigated the impact of 3D-printed models on the understanding, knowledge and anxiety of parents of children undergoing interventional catheterisation.

## Patients and methods

This prospective cross-sectional study was conducted from January 2020 to September 2020 in the Paediatric Cardiology Unit at “Hôpital des Enfants” Toulouse (Toulouse Children's Hospital), France. This tertiary care university hospital is a regional CHD referral centre.

### Study population and intervention

The parents of children undergoing interventional cardiac catheterisation for shunt occlusion were prospectively enrolled in the study. The exclusion criteria included emergency cardiac catheterisations and an inability to understand the questions (language barrier).

Our standard practice for congenital cardiac catheterisations included an in-person preprocedural meeting with the attending congenital interventional cardiologist. During these meetings, the procedure was discussed in detail, with reference to the patient's anatomy. The reason for the procedure was outlined with detailed explanations about the procedure involved. Informed consent was also obtained. Parents were randomly assigned to one of two groups: the printing group or the control group.

**Figure 1:**
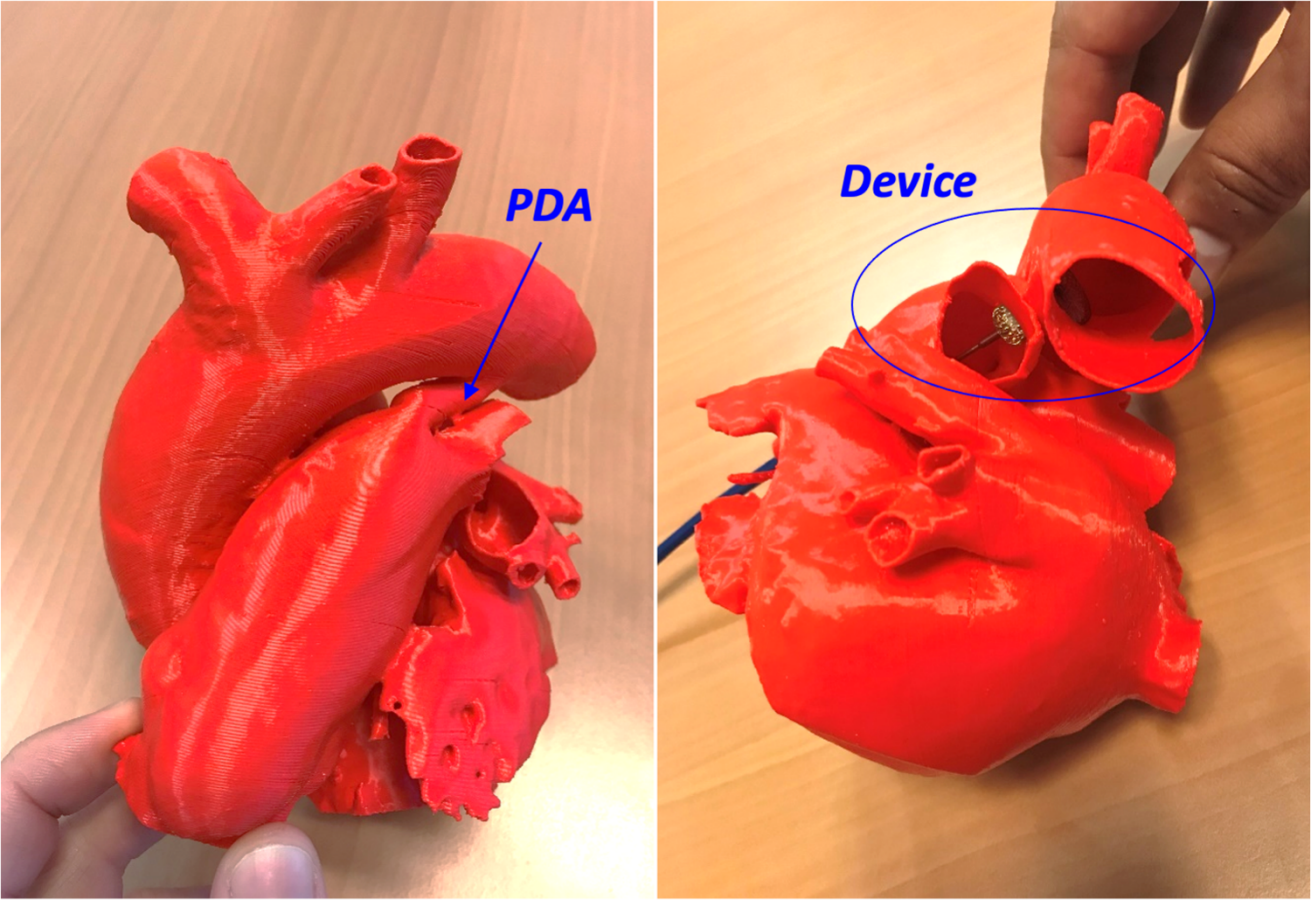
A 3D printed model of patent duct arteriosus (PDA) (left panel), closed with a duct occluder (device) without aortic or pulmonary vascular obstruction as presented during the meeting (right panel).

**Figure 2:**
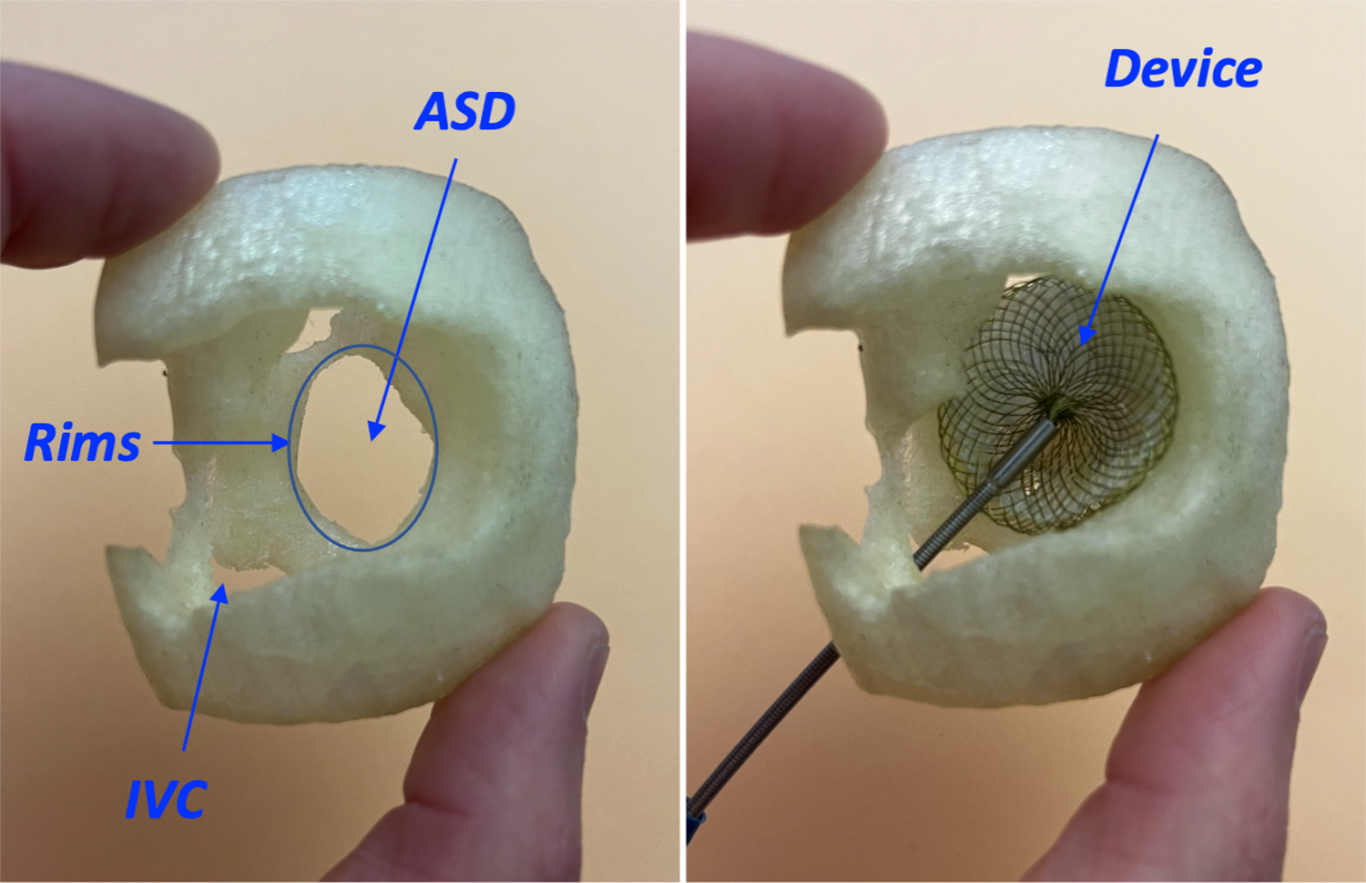
A 3D printed model of atrial septal defect (ASD) from a right atrial view with a sufficient surrounding rims (left panel), closed with an atrial septal occluder (device) without caval obstruction and with good stability as presented during the meeting (right panel). IVC, inferior vena cava.

**Figure 3:**
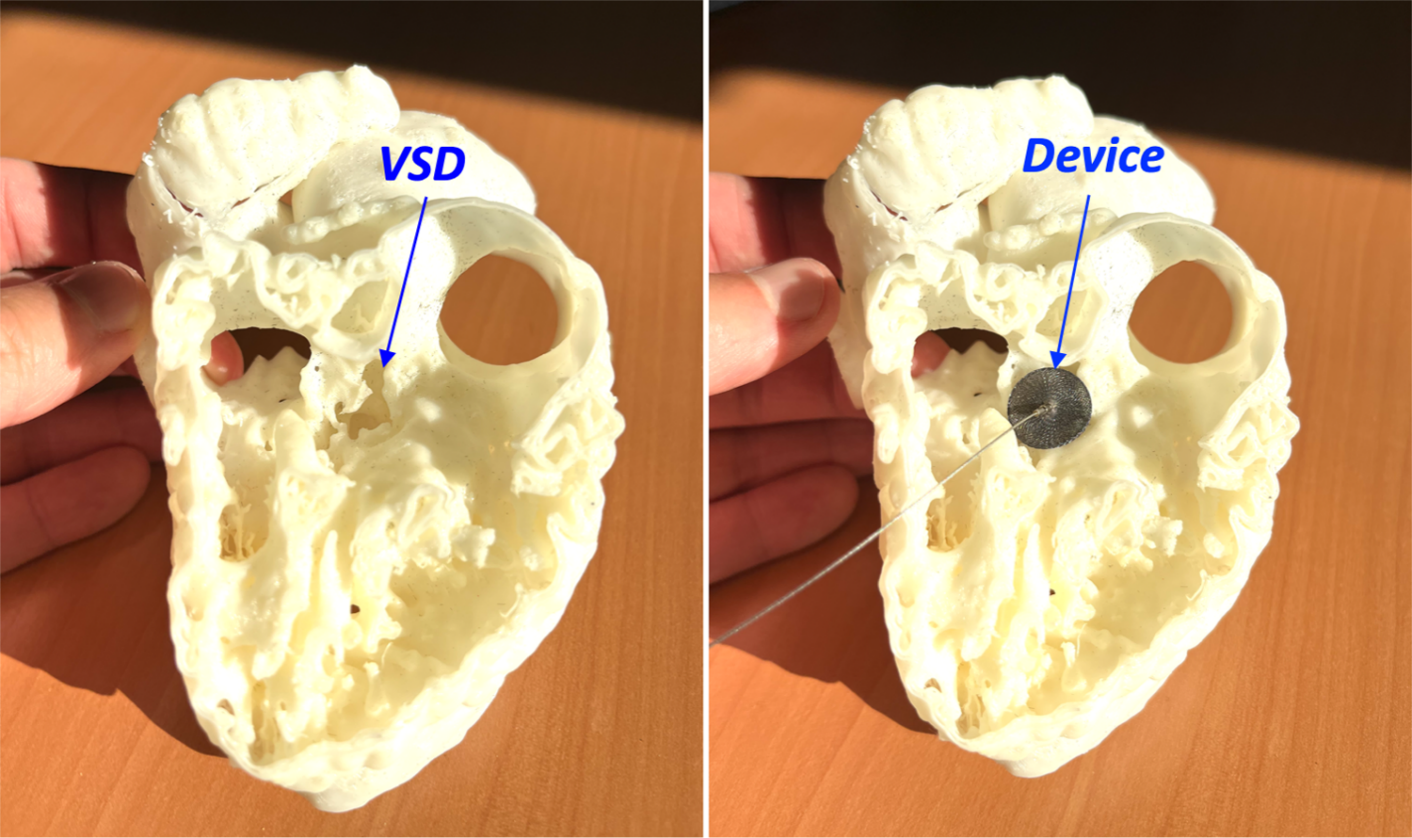
A 3D printed model of perimembranous ventricular septal defect (VSD) from a right ventricle view (left panel), closed with a vascular plug (device) (right panel).

During the meeting, the interventional cardiologist used cardiac diagrams and the device in the control group, and a 3D-printed model of the cardiac lesion with the device in the printing group to give a precise, step-by-step description of the procedure (Figures 1–3).

### Questionnaire assessment

Parents who gave their informed consent to participate completed pre- and post-meeting questionnaires to assess their anxiety levels and examine their knowledge and understanding of CHD and cardiac catheterisation.

Anxiety was assessed using the State-Trait Anxiety Inventory (STAI) before and immediately after the meeting. We used the State Anxiety subscale (STAI Y-A). STAI scores were in the range of 20–80, with higher scores indicating higher levels of anxiety. A normal score was defined as 34–36 for non-psychiatric patients, with scores above 38 indicating significantly elevated anxiety levels (15).

Each parent also completed the same questionnaire twice, before and after the meeting. A 30-point scale was used, including the 5-point Likert scale (where 1 = strongly disagree, 5 = strongly agree). The questions were used to assess individual knowledge and understanding about the disease and cardiac catheterisation. When both parents took part in the study, they were assigned to the same group. The questionnaires were devised by PA, CK, and KH.

The post-meeting questionnaire also included three additional 5-point Likert scale questions to rate individual satisfaction with the use of cardiac 3D-printed models.

An example of the post-meeting questionnaire is provided (Supplementary material).

### Creating 3D-printed models

We conducted a retrospective search for extractable DICOM records of cardiac CT and 3D echocardiography performed in our hospital over the past year as part of routine CHD follow-up. Based on image quality, and following anonymisation, we selected one 3D echocardiography of ostium secundum atrial septal defect (ASD), one CT scan of patent ductus arteriosus, and one CT scan of a peri-membranous ventricular septal defect (VSD) displaying good accuracy (16).

We used Mimics and 3-Matic (Materialise HQ, Leuven, Belgium) software for segmentation and to generate the final scale. The 3D virtual model was exported as an STL file. STL files were finally printed using a Stream 20 pro printer (Volumic, France) and a biodegradable polylactic acid (PLA) filament. For the ASD model based on echocardiography data, the image was acquired by 3D transoesophageal echocardiography in 3D zoom mode using the EPIQ system (version 7C; Philips Medical Systems, Andover, MA, USA) and an X8-2t phased array transducer. The mean time required for model segmentation was approximately 15 min for ASD and PDA, and 30 min for VSD. The mean times taken to print the models were 30 min, 120 min, and 150 min for ASD, PDA, and VSD, respectively.

The study was conducted in accordance with the principles of the Good Clinical Practice protocol and the Declaration of Helsinki. This study was approved by the institutional review board (Comité d'Ethique de la Recherche, Hôtel-Dieu) of the Direction de la Recherche Médicale et Innovation (Medical Research and Innovation Directorate), Hopitaux de Toulouse (Toulouse Hospitals). Informed consent was obtained from all parents.

### Analysis

Quantitative variables were expressed as mean ± standard deviation (SD). Normally distributed continuous variables were compared with *t* tests, while abnormally distributed variables were compared with Mann–Whitney *U* tests. Normality was assessed using the Shapiro–Wilk normality test. Changes in STAI survey scores were compared with *t* tests or Mann–Whitney *U* tests in terms of normality distribution and paired if appropriate. Increases in scores were assessed by subtracting the pre-meeting score from the post-meeting score. A *t*-test was then used to compare the printing group to the control group. Knowledge ratings from the “pre-” and “post”-meeting surveys were analysed using a Wilcoxon matched-pairs and non-paired signed rank test.

*P*-values < 0.05 were deemed statistically significant. The statistical analysis was carried out using GraphPad Prism 9 (GraphPad Software, Inc., San Diego, CA, USA).

## Results

We enrolled 76 parents of 50 children. The mean age of the children undergoing cardiac catheterisation was 6.5 ± 4.8 years. Of the parents, 60% were mothers. The two groups were comparable for each CHD (Table 1).

**Table 1:** Baseline data.

	Total no. of patients (%)	No. in the printing group (%)	No. in the control group (%)
Parents enrolled	76 (100)	38 (50)	38 (50)
Male	30 (40)	15 (40)	15 (40)
Female	46 (60)	23 (60)	23 (60)
Level of education			
No degree	19 (25)	8 (21)	11 (29)
High school degree	11 (14)	3 (8)	8 (21)
Postgraduate	35 (46)	18 (47)	17 (45)
Socio-professional category			
Craftspeople and business managers	5 (7)	2 (5)	3 (8)
Managers and intellectual professions	13 (17)	5 (13)	8 (21)
Intermediate professions	13 (17)	7 (18)	6 (16)
Employees	18 (24)	7 (18)	11 (29)
Workers	7 (9)	5 (13)	2 (5)
No occupation	7 (9)	3 (8)	4 (11)
CHD of their children	76	38	38
ASD	43 (57)	19 (50)	24 (63)
VSD	6 (8)	3 (8)	3 (8)
PDA	27 (35)	16 (42)	11 (29)

ASD, atrial septal defect, CHD, congenital heart disease, PDA, patent ductus arteriosus, VSD, ventricular septal defect.

**Figure 4:**
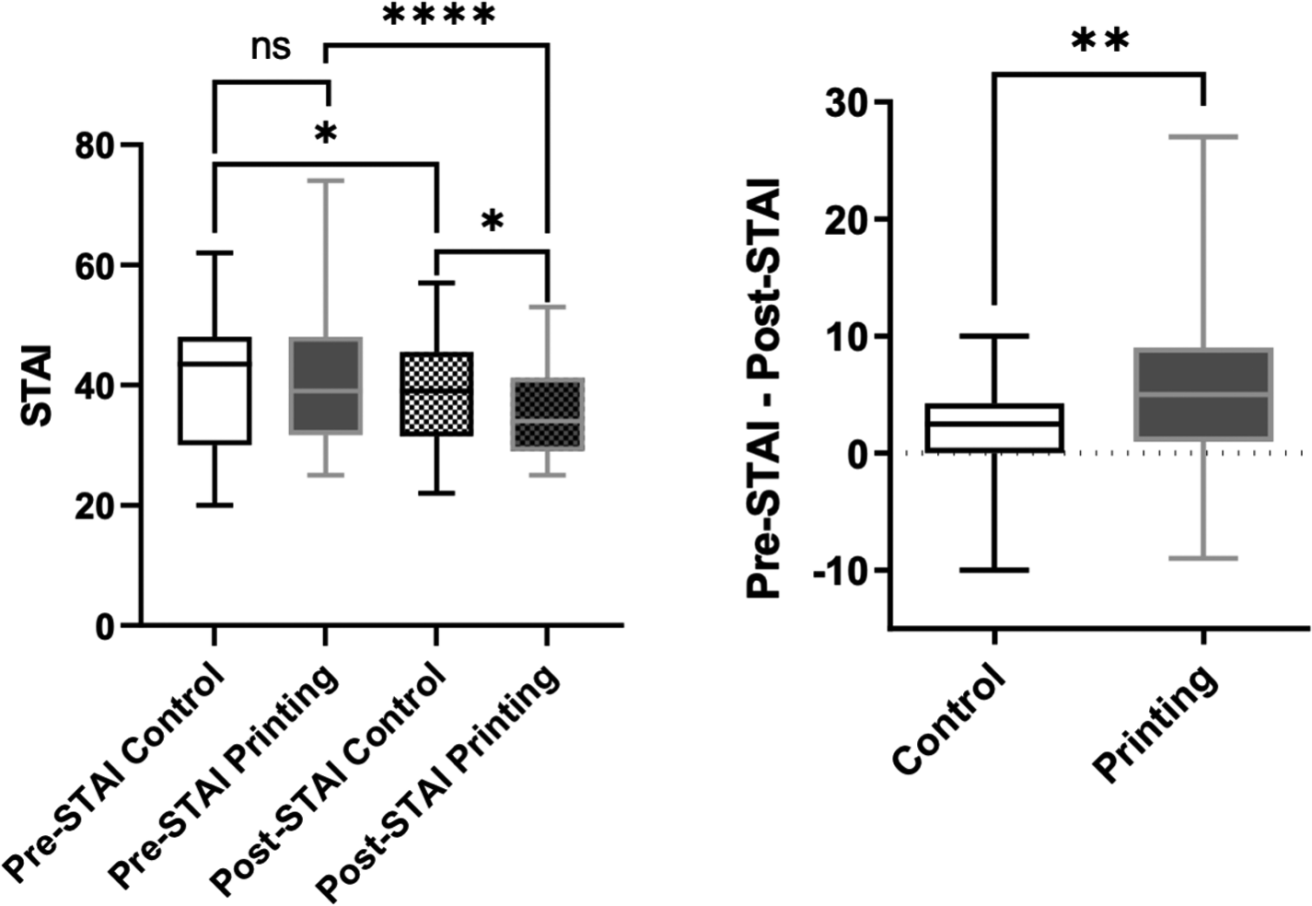
Parent anxiety levels assessed by STAI in the control group compared to the printing group before and after the meeting (left panel). Difference between pre- and post-meeting STAI score in the control group compared to the model group (right panel).

Overall, there was no difference between the printing and control groups in terms of the pre-STAI analysis, with a mean score of 40.9 ± 11.1 out of a minimum of 20 being recorded for the control group and 40.9 ± 11.2 for the printing group (*p* = 0.98) (Figure 4).

**Figure 5:**
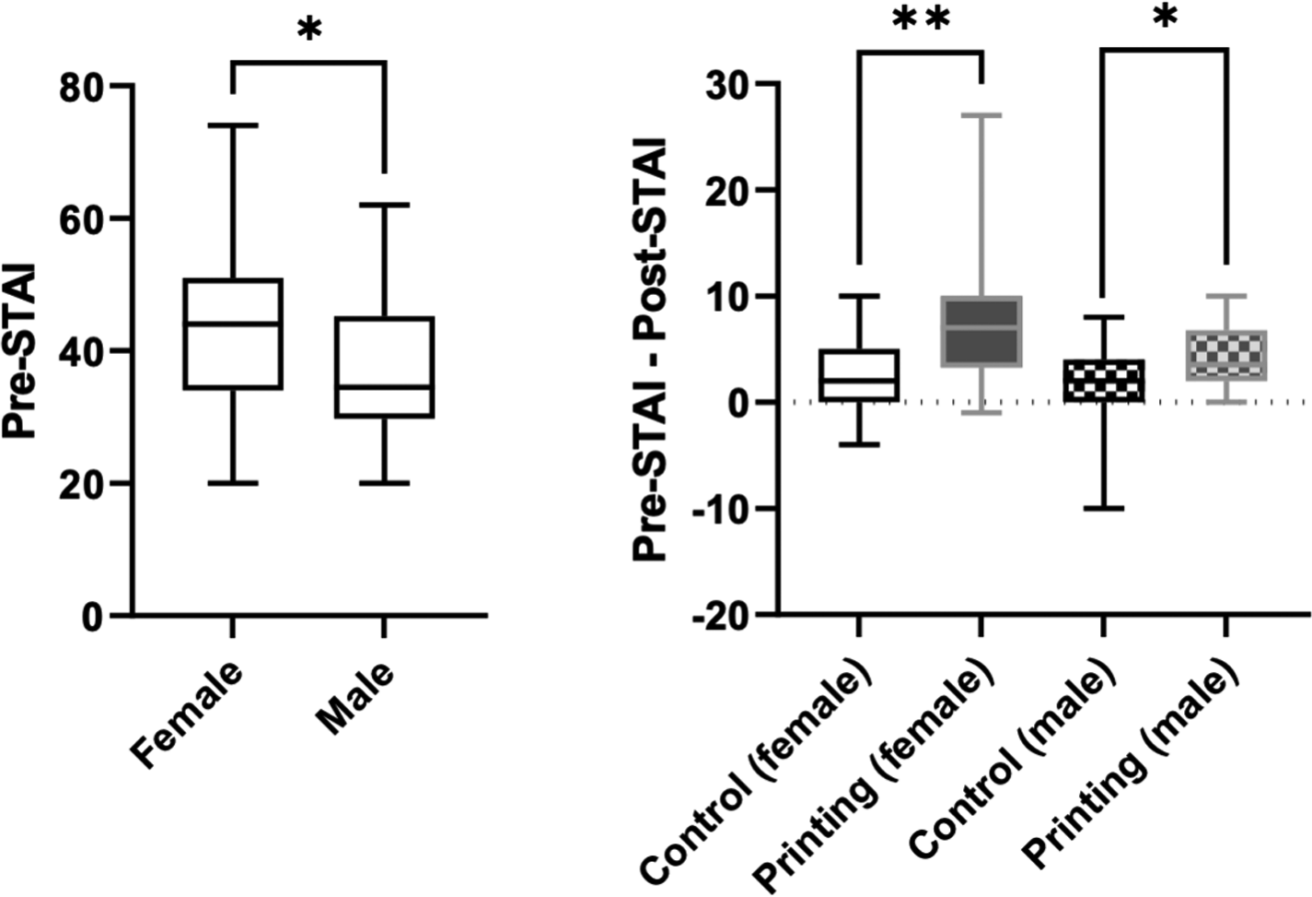
A comparison of anxiety levels in mothers and fathers based on the pre-meeting STAI score (left panel). Differences between pre- and post-meeting STAI scores in the control and printing groups based on parent gender (right panel).

Anxiety levels decreased in both groups after the meeting but remained higher in the control group than the printing group (39.0 ± 9.6 vs. 35.1 ± 7.1, *p* = 0.046). A greater decrease in score was documented in the printing group compared to the control group (+1.9 ± 4.6 vs.  + 5.7 ± 8.0, *p* = 0.006) (Figure 4). At baseline, the mothers were more anxious than the fathers (*p* = 0.02), but the STAI score improved to a greater extent for both the mothers and fathers in the printing group compared to the control group after the meeting (*p* = 0.003 and *p* = 0.03, respectively) (Figure 5).

**Figure 6:**
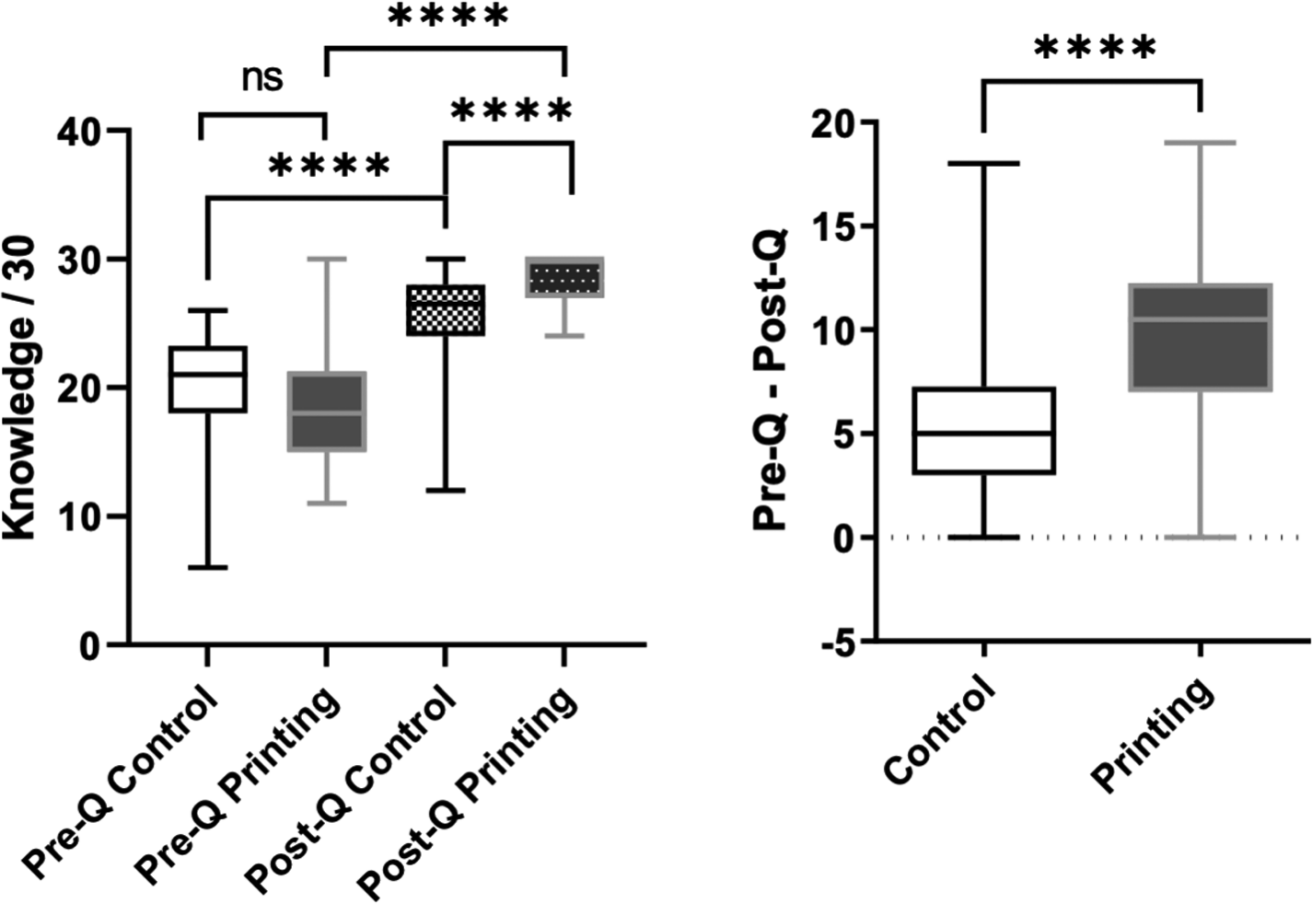
Parental knowledge based on a 30-point scale in the control and printing groups before and after the meeting (left panel). Differences between the pre- and post-meeting knowledge scores in the control and printing groups (right panel).

No difference between the printing and control groups was observed in terms of the pre-meeting score for assessing knowledge, with a mean global score of 20.1 ± 4.5 out of a maximal score of 30 for the control group and 18.3 ± 4.3 out of 30 for the printing group (*p* = 0.08) (Figure 6). The improvement in scores was significantly higher in the printing group compared to the control group ( + 5.4 ± 4.1 vs.  + 10.1 ± 4.3 *p* < 0.0001) (Figure 6).

Most of the parents found the CHD consultation using 3D-printed models to be extremely useful. A score of 4.8 ± 0.5 out of 5 was recorded on the 5-point Likert scale.

## Discussion

Cardiac 3D-printed models improve parents' knowledge and understanding before their child undergoes cardiac catheterisation. More importantly, parental anxiety in the printing group was considerably alleviated after the meeting (Figure 6, middle).

Patient-specific 3D-printed models have a positive effect on clinical decision-making and procedure planning in cardiac surgery or complex cardiac catheterisation (10). In our study, the use of cardiac 3D-printed models increased parental understanding of the child's condition.

One recent study showed that the use of patient-specific 3D-printed models did not significantly improve parental knowledge regarding the child's CHD (14). Nevertheless, the parents found that the use of 3D models in medical consultations was useful in order to understand their child's condition, and the feedback was excellent. An uncontrolled study of 20 adolescent patients with CHD found that the patients' objective knowledge significantly increased following a clinic visit with 3D models (17). Nevertheless, these studies used patient-specific 3D-printed models while we used 3D condition-specific models. Moreover, the use of 3D models as an educational tool is widespread and effective even with 3D condition-specific models (13, 18).

Nowadays, more attention is being paid to the health-related quality of life and psychological well-being of both the children and their parents. Moreover, the physician–patient working alliance is driven by cognitive and psychological dimensions to optimise medical care (19). Our study showed that anxiety levels were high at baseline, particularly in mothers (mean STAI score of 40.9 ± 11.9). A recent study also confirmed that mothers experience significantly greater anxiety than fathers before congenital cardiac surgery (5). Another study involving 203 families referred to an elevated mean STAI score of 38±12 on the day of non-cardiac surgery. Interestingly, some randomised study results showed that parental anxiety levels correlated positively with 'children's anxiety levels (20, 21). In adults, higher levels of anxiety have been linked to increased morbidity and mortality for patients undergoing cardiac procedures (22).

The main finding of our study was the more pronounced decrease in parental anxiety levels thanks to 3D models during the pre-catheterisation meeting (*p* < 0.0001). Some of the parents during the meetings made oral comments that could be attributed to the fact that seeing and touching the model helped to reduce anxiety since parents said they “had a clear idea of the surrounding structure,” and “could feel the device through the defect” or “could imagine how the heart would function with the device.” One pilot study involving 16 parents recorded a decrease in the mean STAI score after the meeting with the cardiologist performing the procedure (39.8 vs. 31.0, *p* = 0.008) (9) and confirmed that 3D-printed models were the most effective tools. Nevertheless, the cohort was small, and the study was conducted without a control group. Other tools have also been shown to reduce anxiety. Playing games, music therapy aimed at distracting participants or entertainment by a clown proved beneficial in reducing anxiety before minor surgery (23–25). A recent meta-analysis highlights the use of videos in providing sufficient information to manage preoperative anxiety in parents (26). It is interesting to note that when children play video games or play with toys before undergoing congenital cardiac surgery, their postoperative stress and anxiety are reduced, and parental anxiety and stress can be alleviated with proper counselling and information (27).

### Limitations

Our study did not assess child anxiety. Nevertheless, the preoperative intervention was more effective at reducing parental as opposed to child anxiety (28). The children in our study also had a broad age range. The data were obtained from completing questionnaires, which could be subject to response, recall and selection bias. However, the STAI assessment is a well-validated tool commonly used to assess patient anxiety and designed to minimise bias from self-reported data (15). We used only STAI Y-A (state anxiety); however, trait anxiety could be considered to be a personality dimension that can be defined as an individual's predisposition to worry or anxiety (state anxiety) (29). Therefore, in future studies, the use of the complete form of STAI could be interesting. The long-term positive impact on knowledge has not been assessed. Regardless of knowledge and anxiety levels, communication with parents and children improved during the meeting thanks to 3D-printed models.

## Conclusion

The 3D-printed model efficiently improves the understanding of disease-related information and parental satisfaction, thereby signiﬁcantly reducing parental anxiety. 3D models improve the knowledge and understanding of parents before their children undergo cardiac catheterisation. Anxiety levels are also reduced. The 3D models should therefore be used as a matter of course during pre-catheterisation meetings.

## Data Availability

The raw data supporting the conclusions of this article will be made available by the authors, without undue reservation.
