# Supplementary material for: Impact of 3D-printed models in meetings with parents of children undergoing interventional cardiac catheterisation
Source: Front Pediatr. 2023 Jan 9;10:947340. doi: 10.3389/fped.2022.947340 (PMC9869040; doi:10.3389/fped.2022.947340)
Supplement: Supplementary file 1 [file Table1.docx]

**Pre-interview questionnaire (1 to each parent) Date ..../..../........**

Child's name ............... Child's first name........ .................

Date of birth ..../..../........

Name of parent .......... .................... First name of the parent........................

Level of study .................... Profession.....................

Check the answer that corresponds to your feelings

|  | Strongly disagree | Rather disagree | Indifferent | Tend to agree | Totally agree |
| --- | --- | --- | --- | --- | --- |
| I know my child's heart disease well | ☐ | ☐ | ☐ | ☐ | ☐ |
| I am able to explain my child's heart disease | ☐ | ☐ | ☐ | ☐ | ☐ |
| I understand how my child's catheterization procedure is going to be | ☐ | ☐ | ☐ | ☐ | ☐ |
| I am able to explain the course of the intervention | ☐ | ☐ | ☐ | ☐ | ☐ |
| I imagine the material that will be put in place and its evolution with the growth of my child | ☐ | ☐ | ☐ | ☐ | ☐ |
| I imagine its evolution with the growth of my child | ☐ | ☐ | ☐ | ☐ | ☐ |
| I am anxious for the procedure my child needs to have | ☐ | ☐ | ☐ | ☐ | ☐ |

Name your child's heart disease: ........................................

**Post-interview questionnaire Patient label**

**Child's name ............... Child's first name........**

**Parent's name .............................. First name of parent.........................**

Check the answer that corresponds to your feelings

|  | Strongly disagree | Rather disagree | Indifferent | Tend to agree | Totally agree |
| --- | --- | --- | --- | --- | --- |
| I know my child's heart disease well | ☐ | ☐ | ☐ | ☐ | ☐ |
| I feel comfortable explaining my child's heart disease | ☐ | ☐ | ☐ | ☐ | ☐ |
| I understand how my child's catheterization procedure is going to be | ☐ | ☐ | ☐ | ☐ | ☐ |
| I feel able to explain the course of the intervention | ☐ | ☐ | ☐ | ☐ | ☐ |
| I imagine the equipment that will be put in place | ☐ | ☐ | ☐ | ☐ | ☐ |
| I imagine its evolution with the growth of my child | ☐ | ☐ | ☐ | ☐ | ☐ |
| I am anxious for the procedure my child needs to have | ☐ | ☐ | ☐ | ☐ | ☐ |
| The 3D printed model was useful to me | ☐ | ☐ | ☐ | ☐ | ☐ |
| The 3D printed model helped me better understand heart disease | ☐ | ☐ | ☐ | ☐ | ☐ |
| The 3D printed model helped me better understand the procedure | ☐ | ☐ | ☐ | ☐ | ☐ |
| The 3D printed model made me anxious | ☐ | ☐ | ☐ | ☐ | ☐ |

Name your child's heart disease: ..............................................
